# Data showing the shapes of cones and Müller cells within the fovea of monkeys reconstructed from serial sections and focused ion beam analysis

**DOI:** 10.1016/j.dib.2018.08.195

**Published:** 2018-09-05

**Authors:** Ulrich Schraermeyer, Sebastian Schmelzle, Alexander V. Tschulakow

**Affiliations:** a Division of Experimental Vitreoretinal Surgery, Center for Ophthalmology, University Hospital Tuebingen, Schleichstr. 12/1, 72072 Tuebingen, Germany; b Ecological Networks, Department of Biology, Technische Universitaet Darmstadt, Darmstadt, Germany; c STZ Ocutox, Burgackerstr. 1, 72379 Hechingen, Germany

**Keywords:** Müller glial cells, Fovea, Cone receptors, Foveola, 3D model, Umbo, Stiles-Crawford effect

## Abstract

The data presented in this article are related to the research paper entitled “The anatomy of the foveola reinvestigated” (Tschulakow et al., 2018) [1]. Here we show the original aligned serial sections through the foveal centre of monkeys at different planes of section and 3 D models of central foveal cells.

**Specifications Table**

**Table:** 

Subject area	*Biology, Medicine*
More specific subject area	*Anatomy of the foveola*
Type of data	*Images, videos*
How data was acquired	*Semithin serial sections from monkey foveae were photographed under a light microscope and aligned by Amira software*
Data format	*Images were aligned, analysed and processed to form 3D models. Image stacks and videos were annotated.*
Experimental factors	*Foveae from monkeys were fixed with 5% glutaraldehyde, embedded in Epon*
Experimental features	*Foveae were serial sectioned in different planes of section with an ultramicrotome*
Data source location	*Münster (Germany), Strasbourg (France)*
Data accessibility	*With this data paper*
Related research article	*A.V. Tschulakow, T. Oltrup, T. Bende, S. Schmelzle, U. Schraermeyer, The anatomy of the foveola reinvestigated. (PeerJ. 2018;6:e4482).*

**Value of the data**

• These data show serial sections through foveal cones and Müller cells of monkeys (Macaca fascicularis).
• The data are valuable for scientists investigating specific features of the macula or fovea in vivo or with histology.
• The data may be helpful for ophthalmologists investigating the pathogenesis of macular telangiectasia type 2.
• The data are helpful for researchers in the field of Müller glial cells.
• The data are valuable for scientists investigating the Stiles-Crawford effect.
• The data help in understanding properties of ocular coherence images of the fovea.

## 1 Data

These data show 21 serial sections through the cones within the foveola of monkeys. The plane of section is parallel to the optic axis. The sections are made at a distance approximately 150 µm from the centre of the foveola. The serial sections are mounted to a video (**Video 1**).

**Video 2**: Here the curved nature of cone inner segments (green) is shown. The outer limiting membrane is labelled dark blue and the nuclear part of the cones light blue. The outer segments (yellow) could only be partly reconstructed because they do not fully fit into this stack of sections. Some outer segments run parallel to the surface of the retinal pigment epithelium (pink).

**Fig. 1:**
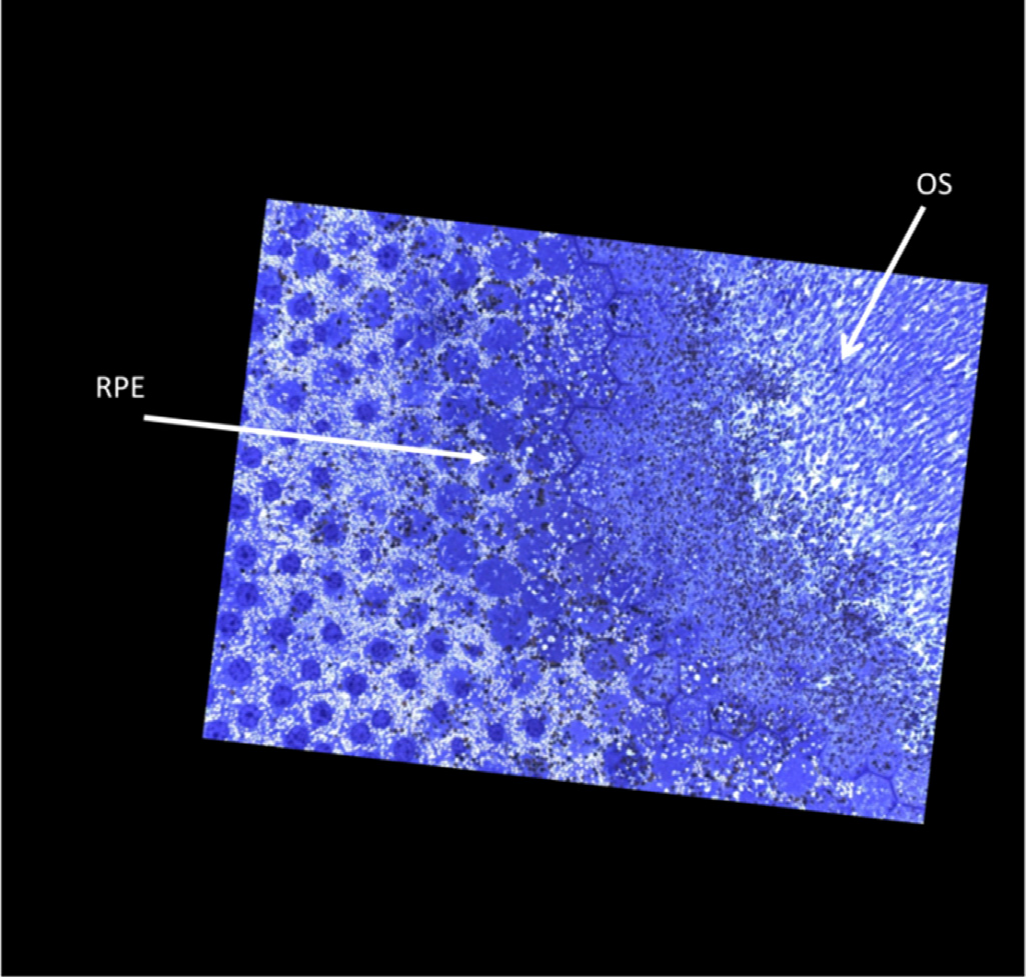
Sections with numbers 1–14 show different levels of the retinal pigment epithelium (RPE). Sections 15–84 shows outer and inner segments (OS, IS) of the foveolar cones. Sections 85–138 contain the outer limiting membrane (OLM). In the centre of section 138 a few cones are a surrounded by the outer limiting membrane (OLM) (dark blue). In sections 139–148 the inner retina of the fovea which contains only two types of cells is shown. Müller cells (MC) appear white and cone photoreceptors appear blue. Thus they can be easily distinguished.

A second series of data comprises 148 sections which run perpendicular to the optic axis. Section 1 is at the level of the retinal pigment epithelium and the series ends within the nuclear layer of the cones (Fig. 1).

Supplementary material related to this article can be found online at doi:10.1016/j.dib.2018.08.195.

The following is the Supplementary material related to this article Video 1, Video 2.

Video 1 On the left the retinal pigment (RPE) is shown. On the right the retina is limited by the inner limiting membrane. The small blue line in the middle represents the outer limiting membrane (OLM). The inner segments (IS) of the cones (to the left of the OLM) are never hit in full length. As they are curved they run out of the plane of section. N depicts the cone nuclei. The inner segments from the cones at this site are curved which is shown in a three dimensional model (Video 2). The outer segments are not in their original position.

Video 2 Here the curved nature of cone inner segments (green) is shown. The outer limiting membrane is labelled dark blue and the nuclear part of the cones light blue. The outer segments (yellow) could only be partly reconstructed because they do not fully fit into this stack of sections. Some outer segments run parallel to the surface of the retinal pigment epithelium (pink)..

**Fig. 2:**
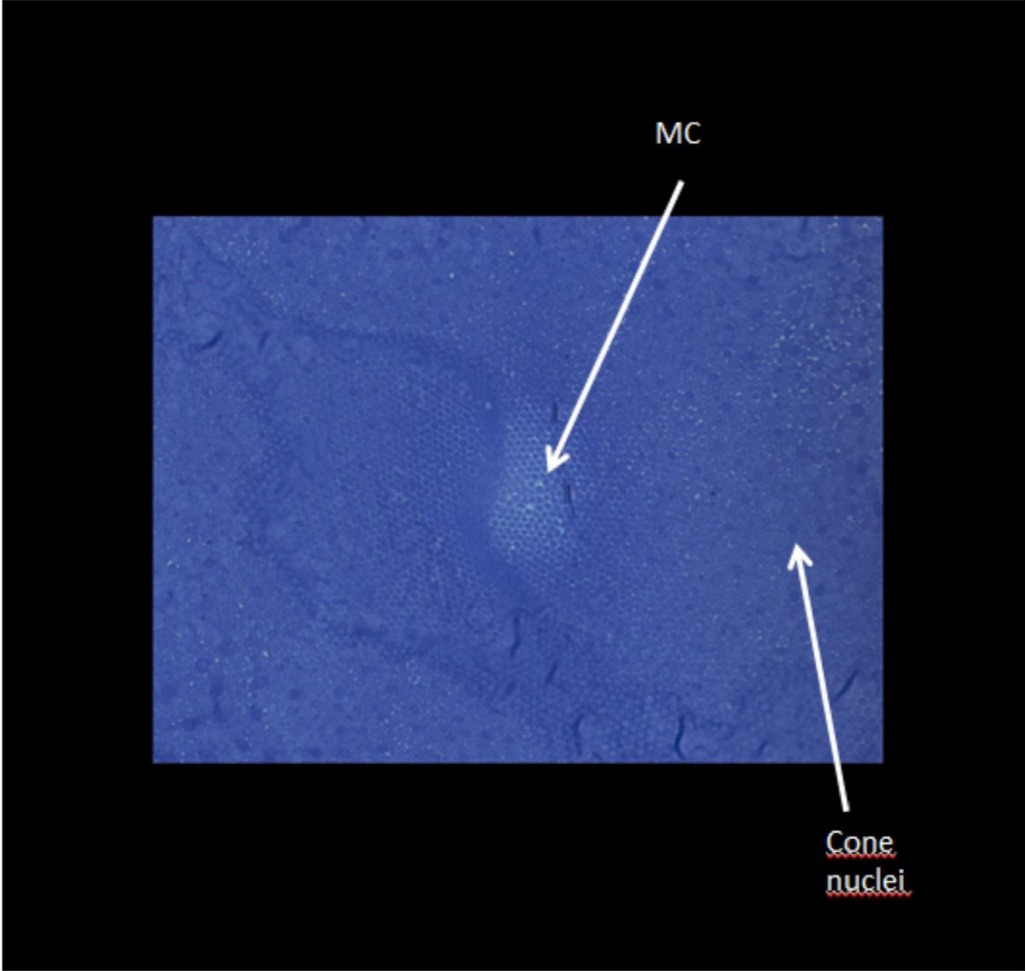
Central foveolar Müller cells (MC) are seen in the foveolar centre. Within the outer retina they do not contain cell organelles and appear white in semithin sections. Surprisingly the shape of the Müller cells is often rectangular or triangular.

A third series of data consists of 49 sections through the retina within the centre of the foveola of a monkey. The plane of section is perpendicular to the optic axis. The series begins at the level of the outer limiting membrane and ends within the nuclear layer of cones (Fig. 2).

**Fig. 3:**
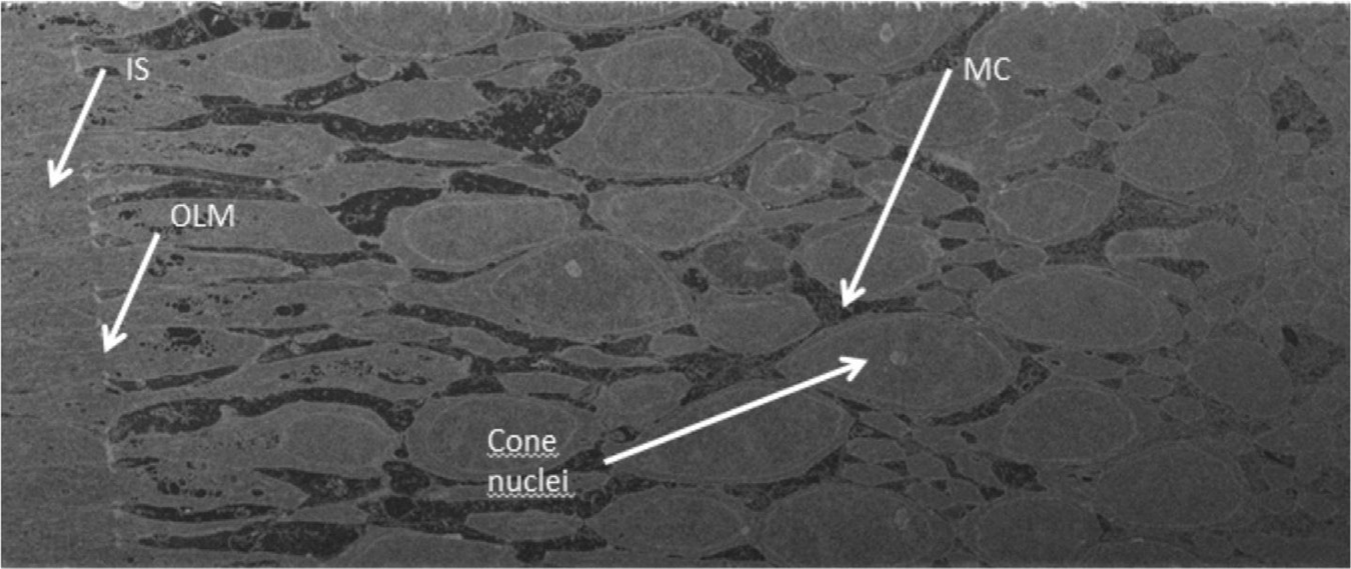
A stack of the central retinal section from a monkey fovea is shown with focused ion beam/scanning electron microscopy tomography. Müller cells (MC) appear electron-dense whereas cones are electron-opaque.

A fourth series was made with focused ion beam microscopy (Fig. 3).

## 2 Experimental design, materials and methods

### 2.1 Light microscopy of monkey eyes

Twenty-four monkey eyes (*Macaca fascicularis*, 14 males, 10 females) were used after the animals were sacrificed under general anaesthetic. Monkeys were kept at Covance Laboratories GmbH Germany and SILABE-ADUEIS, France. For accreditation and details, please see the research paper related to this data. [1] The monkeys were aged between 4 and 8 years. The eyes were enucleated 5 min post-mortem, cleaned of orbital tissue, and were slit at the limbus without causing damage to the ora serrata. The vitreous center was then injected with 200 μl of the fixative (5% glutaraldehyde). The eyes were subsequently fixed and embedded as described [1]. Semithin sections were stained with toluidine blue and examined by light microscopy (Zeiss Axioplan 2 imaging, Zeiss, Jena, Germany).

### 2.2 Evaluation of serial sections through the fovea of monkeys

The 3D model reconstruction was created by photographing semithin serial sections taken from monkey foveae.

### 2.3 Three dimensional modelling

The 3D reconstruction of the figures and measurements presented here was carried out using Amira^®^ software (version 5.6; FEI, Hillsboro, Oregon, USA). After digitalization, the section images were aligned manually by comparing superimposed slices, translating, and rotating adjacent slides in relation to one another. In addition, the border of each slide as well as regular patterns and structure in the slides were used as markers for alignment. The aligned sections were then imported into the Amira Software, and the relevant structures labelled using the software segmentation tools.

### 2.4 Focused ion beam/scanning electron microscopy

Focused ion beam/scanning electron microscopy (FIB/ SEM) tomography data were obtained using a Zeiss Auriga CrossBeam instrument at the Natural and Medical Sciences Institute at the University of Tuebingen (NMI; Reutlingen, Germany) as described [2].

In brief, the embedded sample block was sputter coated with gold palladium and mounted on to an SEM sample holder. A light microscope was used to image a semithin section of the embedded sample, and this was then correlated with the SEM image of the ultramicrotome block face to determine the area of interest for three-dimensional analysis. FIB/SEM serial sectioning tomography was performed using a Crossbeam instrument (Zeiss) equipped with a gallium FIB and a low voltage SEM. In this way, the gallium FIB produces a series of cross-sections showing the area of interest.

Each cross-section was imaged by low keV SEM using the energy-selected backscattered (EsB) electron detector for image capture. For full details see [1].

For FIB, the following parameters were used: Primary energy of 30 keV, slicing was carried out with a probe current of 2 nA, the slice thickness was 42 nm. In this way cubic voxels were obtained, i.e. the same resolution in x, y, and z, which is expedient for the reconstruction. The resulting stack of two-dimensional images was used for three-dimensional reconstruction using the relevant software.

## References

[1] Tschulakow A.V., Oltrup T., Bende T., Schmelzle S., Schraermeyer U. (2018). The anatomy of the foveola reinvestigated. Peer J..

[2] Steinmann U., Borkowski J., Wolburg H., Schroppel B., Findeisen P., Weiss C. (2013). Transmigration of polymorph nuclear neutrophils and monocytes through the human blood-cerebrospinal fluid barrier after bacterial infection in vitro. J. Neuroinflammation.

